# Diethyl Aminoethyl Hexanoate Increase Relay Strip Intercropping Soybean Grain by Optimizing Photosynthesis Aera and Delaying Leaf Senescence

**DOI:** 10.3389/fpls.2021.818327

**Published:** 2022-01-05

**Authors:** Kai Luo, Xiaoting Yuan, Chen Xie, Shanshan Liu, Ping Chen, Qing Du, Benchuan Zheng, Yushan Wu, Xiaochun Wang, Taiwen Yong, Wenyu Yang

**Affiliations:** ^1^College of Agronomy, Sichuan Agricultural University, Chengdu, China; ^2^Sichuan Engineering Research Center for Crop Strip Intercropping System / Key Laboratory of Crop Ecophysiology and Farming System in Southwest, Ministry of Agriculture, Chengdu, China

**Keywords:** plant growth regulator (PGR), soybean (*Glycine max* L. Merr.), maize-soybean relay strip intercropping system, diethyl aminoethyl hexanoate (DA-6), soybean yield, leaf senescence, grain filling

## Abstract

Insufficient and unbalanced biomass supply inhibited soybean [*Glycine max* (L.) Merr.] yield formation in the maize-soybean relay strip intercropping (IS) and monoculture soybean (SS). A field experiment was conducted to explore the soybean yield increase mechanism of DA-6 in IS and SS treatments. In this 2-year experiment, compact maize “Denghai 605” and shade-tolerant soybean “Nandou 25” were selected as cultivated materials. DA-6 with four concentrations, i.e., 0 mg/L (CK), 40 mg/L (D40), 60 mg/L (D60), and 80 mg/L (D80), were sprayed on soybean leaves at the beginning of flowering stage of soybean. Results showed that DA-6 treatments significantly (*p* < 0.05) increased soybean grain yield, and the yield increase ratio was higher in IS than SS. The leaf area index values and net photosynthesis rate of IS peaked at D60 and were increased by 32.2–49.3% and 24.1–27.2% compared with the corresponding CK. Similarly, DA-6 treatments increased the aboveground dry matter and the amount of soybean dry matter accumulation from the R1 stage to the R8 stage (VDM_T_) and highest at D60 both in IS and SS. D60 increased the VDM_T_ by 29.0–47.1% in IS and 20.7–29.2% in SS. The TR_*G*_ at D60 ranged 72.4–77.6% in IS and 61.4–62.5% in SS. The MDA content at D60 treatment was decreased by 38.3% in IS and 25.8% in SS. The active grain-filling day in IS was about 7 days longer than in SS. In D60 treatment, the V_mean_ and V_max_ increased by 6.5% and 6.5% in IS and 5.7% and 4.3% in SS compared with the corresponding CK. Although the pod number and hundred-grain weight were significantly (*p* < 0.05) increased by DA-6 treatments, the grains per pod were maintained stable. The pod number and hundred-grain weight were increased by 30.1–36.8% and 4.5–6.7% in IS and 6.3–13% and 3.6–5.6% in SS. Thus, the grain yield at D60 was increased by 36.7–38.4% in IS and 21.7–26.6% in SS. DA-6 treatments significantly (*p* < 0.05) increased soybean grain yield and peaked D60 treatments both in IS and SS.

## Introduction

Soybean (*Glycine max* L. Merr.), a vital legume economic crop with high protein concentration and rich nutritional value, has been widely planted worldwide (Baghdadi et al., 2016). The unbalance between the increasing soybean demand and population expansion and the limited cultivable land area has imperiled the food security of the world (Kai et al., 2020). Intercropping systems, two or more crops planted on the same farmland, have many advantages such as better exploitation of sunlight, water, nutrients, and other environmental resources and higher land equivalent ratio (Li B. et al., 2020). The IS increases the compensation effect of gramineous and leguminous intercropping (Li S. et al., 2020), improves resource utilization in the spatiotemporal niche (Ahmed et al., 2020), increases farmland productivity with less environmental costs, and provides a potential approach to achieving sustainable agriculture development (Darch et al., 2018). This system produced additional soybean yields when maintaining the stability of maize yield production (Chen et al., 2019). However, the higher maize shading during the co-growth period inhibited the vegetative growth and yield formation of relay intercropped soybean (Raza et al., 2019b).

Soybean is sensitive to the changes in light quantity and quality, especially during germination to flowering stage (Yang et al., 2014). The long period of maize shading decreased the PAR) and red/far-red (R:FR) ratio of the soybean canopy, which decreased the soybean carbohydrate synthesis and accumulation and wreaked the vegetative soybean growth (Ahmed et al., 2018). The maize shading inhibited soybean growth and aggravated the resource competition between vegetative and reproductive organs, increasing flower, and pod abortion and abscission (Ahmed et al., 2018). The change in the leave photosynthesis process directly influenced the soybean assimilate supply availability and determined the soybean yield formation processes (Yang et al., 2018; Raza et al., 2019a). Although after maize was harvested, with the recovery of soybean canopy illumination, the growth of relay intercropped soybean was compensated and the yield of relay intercropped soybean was lower than monoculture soybean (Yang et al., 2014). Improving the photosynthesis capacity of the soybean leaf after the flowering stage is an efficient way to maintain the high assimilate supply levels and increase soybean grain yield in the maize-soybean relay strip intercropping and monoculture systems.

Reasonable agricultural practices, such as applying chemical additives, microorganisms, and agronomic practices, could improve crop photosynthetic capacity and carbon-nitrogen metabolism to increase grain yield production (Ba et al., 2020). Diethyl aminoethyl hexanoate is an artificial tertiary amine plant growth regulator with low molecular weight and highly bioactive, which promotes germination and seedling establishment from aged soybean seeds by enhancing the hydrolysis of triacylglycerol and the conversion of fatty acids to sugars (Zhou W. et al., 2019). DA-6 belongs to the cytokines (CKs), which enhance chlorophyll and nucleic acid levels, thus stimulating photosynthesis and cell division (He et al., 2014). It is widely applied in maize (Qi et al., 2013), soybean (Liu et al., 2019), wheat (Wen et al., 2019), and microalgae (Salama et al., 2017) due to its excellent characteristics of environmentally friendly and efficient production enhancement. Spraying DA-6 on soybean at the seeding growth and flowering stages increases soybean pod setting by promoting soybean photosynthesis performance and regulating sucrose and starch metabolism (Qi et al., 2013; Liu et al., 2019). Our previous studies proved that the foliar application of the DA-6 on soybean at the flowering stage performs better on increasing soybean yield than the application of 6-benzylaminopurine and uniconazole (Luo et al., 2020). However, the mechanisms of DA-6 regulate the photosynthetic function and material accumulation and distribution to increase the yield of relay intercropped soybean is still unclear.

With that goal in mind, we hypothesized that DA-6 improved soybean photosynthesis capacity and promoted biomass translocation into grains, promoting the soybean after-flower growth and increasing soybean grain yield, and the yield increase mechanisms in IS and SS patterns were different. Therefore, the aim of this study was to investigate the effects of DA-6 with different patterns on soybean yield and yield components, to evaluate the impact of DA-6 on leaf photosynthesis capacity and pod formation in two patterns, and to investigate the association between leaf senescence and grains formation in different patterns after spraying DA-6. Explore the yield increase mechanisms of DA-6 in soybean could promote the application of DA-6 in maize-soybean relay strip intercropping system.

## Materials and Methods

### Plant Material and Growth Conditions

**FIGURE 1:**
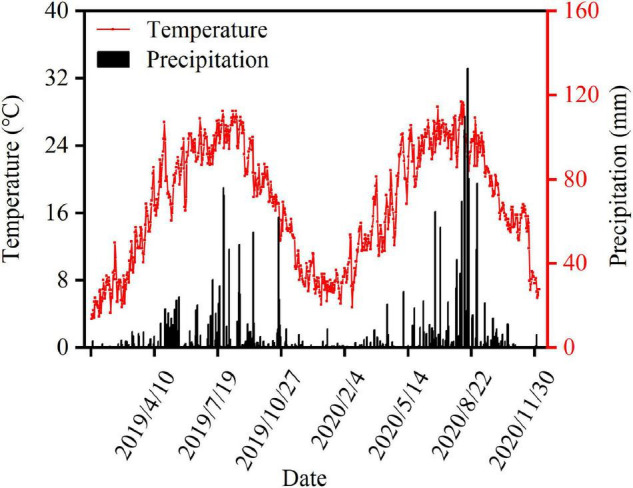
Daily air temperature and precipitation during the maize and soybean growing period in Chongzhou in 2019 and 2020.

A field experiment was conducted between 2019 and 2020 in Chong’zhou Modern Agricultural Research and Development Base, Sichuan Province, China. The field soil was a light loam with 7.42 pH, 26.25 g kg^–1^ organic matter, 1.25 g kg^–1^ total nitrogen (N), 95.03 mg kg^–1^ available potassium, and 16.47 g kg^–1^ available phosphorus. Meteorological data were recorded by a weather station situated at the experimental site (Figure 1). Nanchong Academy of Agricultural Sciences in Sichuan Province and Shandong Denghai Seeds Co. Ltd provided the shade-tolerant soybean “Nan Dou 25” (ND25) and compact maize “DengHai 605,” respectively. Sangon Biotech (Shanghai) Co. Ltd provided the plant growth promoter DA-6 (purity ≥ 98%).

The experiment used a two-factor split-plot experiment design with three replications. Factor A was planting patterns, i.e., IS and SS; Factor B was four DA-6 concentration treatments i.e., including 0 mg/L (CK), 40 mg/L (D40), 60 mg/L (D60), and 80 mg/L (D80). DA-6 was sprayed on soybean leaves at the soybean flowering stage with a spraying dose of 450 L ha^–1^. DA-6 was sprayed on two sides of soybean leaves at 4:00–6:00 p.m. on a sunny and no wind day.

The IS pattern used wide-narrow row planting, where the narrow row spacing of maize was 40 cm, the wide row spacing was 160 cm, two rows of soybean planted in the wide row, the row spacing in the soybean strip was 40 cm, the spacing between maize and soybean was 60 cm, the bandwidth was 2 m, and the plots measured 6 m × 6 m (Chen et al., 2019). Maize was sowed with 17 cm hole distance and 58,500 plants ha^–1^ of density on April 15, 2019, and April 7, 2020, respectively. Soybean was sowed with 8.5 cm hole distance and 117,000 plants ha^–1^ of density on June 19, 2019, and June 15, 2020. The chemical fertilizer amount for soybean were P_2_O_5_ 63 kg ha^–1^ and K_2_O 52.5 kg ha^–1^ and for maize were 240 kg N ha^–1^, P_2_O_5_ 105 kg ha^–1^, and K_2_O 112.5 kg ha^–1^. All P and K fertilizers for crops were used as base fertilizers. The N for maize was divided into base fertilizer (120 kg N ha^–1^) and topdressing (120 kg N ha^–1^) at the maize anthesis-silking stage.

### Soybean Leaf Area Parameters

At the beginning flowering stage (R1), the beginning of pod stage (R3), and the beginning of seed stage (R5), three similar soybeans in each plot were continuously selected to measure the leaf area parameter of soybean by the scanning pixel method described by O’Neal et al. (2002), then the soybean LAI (the one-sided proportion of leaf area per unit ground area) was calculated.

### Soybean Leaf Chlorophyll Content and Net Photosynthesis Rate

Third upmost fully expanded leaves of three soybeans were selected in each plot to measure the soybean chlorophyll content at the R1, blooming flower stage (R2), R3, blooming pod stage (R4), and R5 stages. Chlorophyll pigments were extracted by grinding 0.1 g leaves in 10 ml 80% acetone in the dark at room temperature. Then the spectrophotometer (DU-730; BeckMan Coulter, California, United States) was used to measure the absorbance of the solution at wavelengths of 649 and 665 nm. The chlorophyll content was calculated by the method described by Yao et al. (2017).

Five similar soybean plants were selected in each plot for long-term investigation of the soybean photosynthesis parameters at the R1, R2, R4, and the grain-filling stages (R6). The photosynthesis parameters of third upmost fully expanded leaves of the soybeans were measured between 9:00 and 11:00 a.m. on a sunny day by using the portable photosynthesis system (LI-6400XT; LI-COR Inc., Lincoln, NE, United States). The leaf chamber settings were 1,000 mol m^–2^ s^–1^ photon flux density, 500 μmol s^–1^ flow rate, and 30°C leaf temperature as described by Luo et al. (2020).

### Soybean Leaf Reactive Oxygen and Antioxidant Enzymes Activity

At the R4 and R6 stages, third upmost fully expanded leaves of three plants in each plot were immediately frozen in liquid nitrogen. All the spectrophotometric analyses were conducted using the spectrophotometer (DU-730; BeckMan Coulter, California, United States). The activities of the enzymes of superoxide dismutase (SOD), peroxidase (POD), catalase (CAT), and the content of superoxide anion (O_2_^–^), hydrogen peroxide (H_2_O_2_), and malondialdehyde (MDA) were measured using the assay kit (Solarbio, Beijing, China) according to the description of the manufacturer.

Glutathione reductases activity was determined by the method described by Xu et al. (2016), where absorbance change at 340 nm caused by NADPH oxidation was recorded. APX activity was determined by measuring the change in absorbance at 290 nm as the method described by Jin et al. (2008). The content of glutathione (GSH) and oxidized glutathione (GSSG) was measured using the assay kit (Solarbio) which used the 5,5’-Dithiobis- 2-nitrobenzoic acid ss (DTNB)-GR method to measure the absorbance at 412 nm according to the description of the manufacturer.

### Soybean Dry Matter Distribution and Grain-Filling Process

Three similar plants were sampled in each plot at the R1, R3, R5, and the fully mature (R8) stages to measure the weight of the soybean aboveground dry matter. The soybean from the cotyledon scar were cut and the plants were divided into different organs (including stem, leaf, and pod), then dried the plants at a constant temperature of 80°C after 105°C inactivation for 30 min, eventually the biomass weight on an electronic balance was recorded.

The dry matter distribution index was calculated as described by Luo et al. (2020) using the following formula. DM_A_ was the soybean total dry matter accumulation from the R1 stage to the R8 stage. VDM_T_ was the amount of soybean dry matter transferred into grains from the R1 stage to the R8 stage. TR was the dry matter transportation ratio of VDM_T_ to the dry matter of the vegetative organ at the R4 stage. TR_G_ was the grains contribution ratio of VDM_T_.


(1) $$D\,M_{A}=D\,M_{R\,8}-D\,M_{R\,1}$$

(2) $$V\,D\,M_{T}=V\,D\,M_{R\,4}-V\,D\,M_{R\,8}$$

(3) $$TR\left(\%\right)=\frac{V\,D\,M_{T}}{V\,D\,M_{R\,4}}\times 100\%$$

(4) $$TR_{G}\left(\%\right)=\frac{V\,D\,M_{T}}{Y_{R\,8}}\times 100\%$$

where, DM_*R*1_ and DM_*R8*_ were the soybean dry matter at the R1 and R8 stages. VDM_*R4*_ and VDM_*R8*_ were the dry matter of the soybean vegetative organ at the R4 and R8 stages. Y_*R8*_ was the soybean grain yield at the R8 stage.

The soybean grain-filling process was investigated from 40 days after soybean flowering to the full maturity stage (R8), at a 7-day interval, where the soybean grains were separated from pod and then the constant weight after drying the soybeans at a suitable temperature was recorded. Using the formula *W* = A/(1 + *Be^−kt^*) to fit the soybean grain-filling process, the grain-filling parameters were calculated using the derived formula described by He et al. (2020), where, the *W* (mg) is the grain weight, *A* (mg) is the upper asymptote of the grain weight, *B* and *k* are the coefficients determined by the curvature, and *t* (day) is the day after anthesis. Then, the grain-filling process parameters were calculated using the following formula. *T*_max_ was the time required to reach the maximum grain-filling rate. *T* was the duration time when *W* was achieved at the 99% of A. *V*_max_ and *V*_*mean*_ were the maximum and mean grain-filling rates, and *W*_max_ was the grain weight at maximum grain-filling rate. D was the active grain-filling period and defined as the period when *W* was between 5% to 95% of A.


(5) $$T_{m\,a\,x}=\frac{\text{LnB}}{\text{k}}$$

(6) $$T=\frac{\left(\mathrm{LnB}+4.59512\right)}{\text{k}}$$

(7) $$w_{m\,a\,x}=\frac{\left(\text{A}\right)}{2}$$

(8) $$v_{m\,e\,a\,n}=\frac{W}{1+B\,e^{-k\,t}}/T$$

(9) $$v_{m\,a\,x}=\text{K}\times W_{m\,a\,x}\times \left(1-\frac{W_{m\,a\,x}}{A}\right)$$

(10) $$D=\frac{6}{K}$$

### Soybean Yield and Yield Components

At the full maturity stage, 15 consecutive plants were randomly selected to measure the yield components of soybeans, including the number of pods and grains per plant and hundred-grain weight. The whole soybean plants of 6 m long strip were collected to measure the actual yield of soybean when the water content of the seed was about 13.5% after threshing and drying to record the weight and calculate the yield per hectare.

### Statistical Analyses

The normality of data distribution and the uniformity of variance were tested using the Shapiro-Wilk test and Levine’s test. The differences between treatments were examined using the analysis of variance (ANOVA) followed by the Fisher’s test, and the significant differences were considered at *p* < 0.05 level. The data analyses and picture drawing used Excel software (Microsoft Office Standard 365, Microsoft, Redmond, WA, United States) and Origin Pro 2021 (Learning version) (Origin Lab., Hampton, MA, United States).

## Results

### DA-6 Increases Soybean Photosynthesis Area and Net Photosynthesis Rate

**FIGURE 2:**
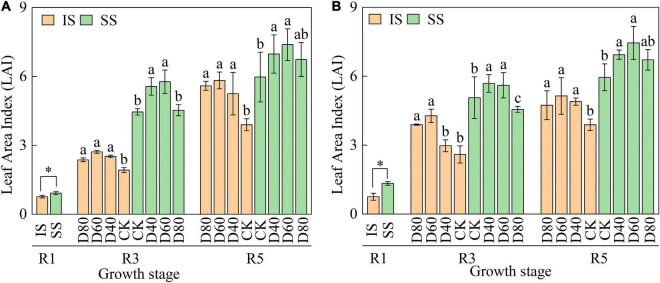
Spraying DA-6 affects the total leaf area of soybean in the 2019 and 2020 growing seasons. **(A,B)** On the right corner of the picture represent the 2019 and 2020 growing seasons, respectively. The bar and error bars represent the mean soybean leaf area of three replicates and the standard deviation of the mean, respectively. Different letters indicate significant differences between treatments at 0.05 probability levels by Fisher LSD multiple comparisons in the same planting pattern. * indicates statistical significance at 0.05 probability levels (*p* < 0.05).

Although the LAI continuously increased from the R1 to R5 stages, LAI was higher in SS than in IS (Figure 2). Importantly, LAI was significantly (*p* < 0.05) enhanced by DA-6 treatments, and the LAI peaked at D60 in both IS and SS. In D60 treatment, the LAI was increased by 32.2–49.3% in IS and 23.6–25.2% in SS compared with the corresponding CK.

**FIGURE 3:**
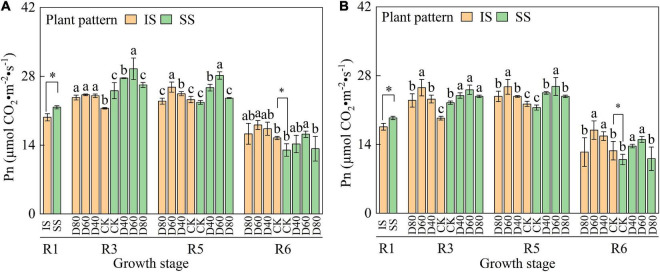
Spraying DA-6 affects the net photosynthesis rate of soybean in the 2019 and 2020 growing seasons. **(A,B)** On the right corner of the picture represent the 2019 and 2020 growing seasons, respectively. The bar and error bars represent the mean soybean leaf area of three replicates and the standard deviation of the mean, respectively. Different letters indicate significant differences between treatments at 0.05 probability levels by Fisher LSD multiple comparisons in the same planting pattern. * indicates statistical significance at 0.05 probability levels (*p* < 0.05).

The Pn value increased and then decreased with the advance of growth periods, and the Pn value in SS was higher than in SS from the R1 to R5 stages (Figure 3). Pn was significantly (*p* < 0.05) increased by DA-6 treatments, and the Pn peaked at D60 in both IS and SS. At the R6 stage, Pn was significantly higher in IS than in SS. In D60 treatment, the Pn was increased by 24.1–27.2% in IS and 17.1–19.8% in SS compared with the corresponding CK.

### DA-6 Enhances the Activity of Soybean Antioxidant Metabolism

**FIGURE 4:**
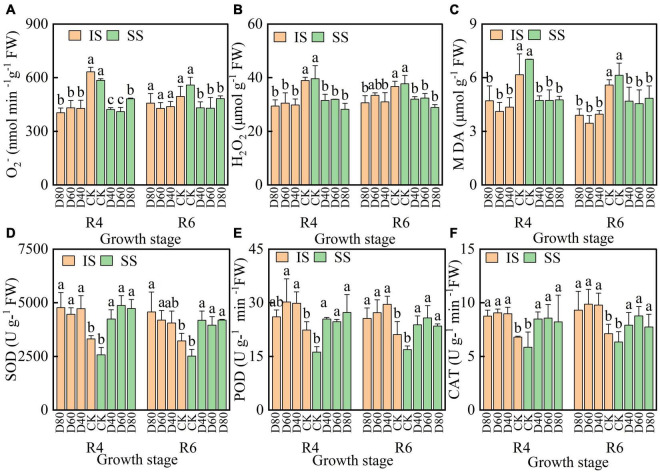
Spraying DA-6 affects reactive oxygen species and membrane lipid peroxidation products content, and the SOD, CAT, and POD enzymes activities of soybean. **(A–C)** O_2–_, H_2_O_2_ and MDA content in soybean leaves. **(D–F)** enzymes activity of SOD, POD, and CAT in soybean leaves. The bar and error bars represent the mean soybean leaf area of three replicates and the standard deviation of the mean, respectively. Different letters indicate significant differences between treatments at 0.05 probability levels by Fisher LSD multiple comparisons in the same planting pattern.

The values of O_2_^–^ and H_2_O_2_ were decreased by DA-6 treatments, O_2_^–^ and H_2_O_2_ values were lower in IS than in SS (Figures 4A–C). The MDA value was significantly (*p* < 0.05) decreased by DA-6 treatments, and the Pn value was lowest at D60 in both IS and SS. At the R6 stage, D60 significantly decreased the MDA values by 38.3% in IS and by 25.8% in SS compared with the corresponding CK.

The values of SOD, POD, and CAT were increased by DA-6 treatments, and the SOD, POD, and CAT values at CK were higher in IS than in SS (Figures 4D–F). The SOD values were highest at D80 and D60 in IS and SS, respectively. The values of POD and CAT were significantly enhanced by DA-6 treatments and peaked at D60 in both IS and SS.

### DA-6 Improves Soybean Biomass Distribution and Grain-Filling Processes

**FIGURE 5:**
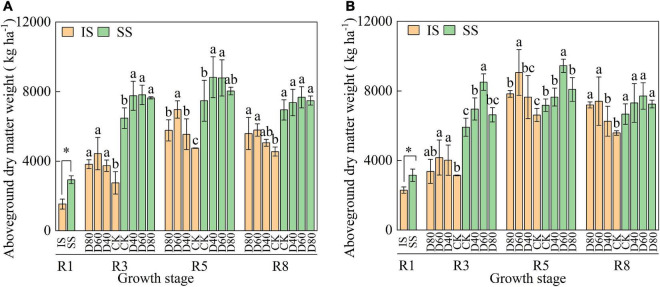
Spraying DA-6 affects the aboveground dry matter weight of soybean in the 2019 **(A)** and 2020 **(B)** growing seasons. The bar and error bars represent the mean soybean leaf area of three replicates and the standard deviation of the mean, respectively. * indicates statistical significance at 0.05 probability levels (*p* < 0.05). Different letters indicate significant differences between treatments at 0.05 probability levels by Fisher LSD multiple comparisons in the same planting pattern.

Aboveground dry matter increased from the R1 stage and peaked at the R5 stage, and aboveground dry matter was higher in SS than in IS (Figure 5). DA-6 treatments significantly (*p* < 0.05) increased aboveground dry matter and peaked at D60 in both IS and SS. In D60 treatment, at the R8 stage, the aboveground dry matter was increased by 27.2–32.6% in IS and 8.5–15.6% in SS compared with the corresponding CK.

The values of DM_A_, VDM_T_, TR, and TR_G_ were increased by DA-6 treatments (Table 1). DM_A_, VDM_T_, TR, and TR_G_ values were higher in IS than in SS. In D60 treatment, the DM_A_ and VDM_T_ values were increased by 34.2–51.4% and 29.0–47.1% in IS, and increased by10.0–32.5% and 20.7–29.2% in SS, compared with the corresponding CK. The values of TR and TR_G_ were peaked at D60 in both IS and SS. In D60 treatment, the TR and TR_G_ values reached 54.1–55.4% and 72.4–77.6% in IS.

**TABLE 1:** Spraying DA-6 affects the dry matter accumulation and distribution of soybean after anthesis.

Year	Plant pattern	Treatment	DM_A_ (kg ha^–1^)	VDM_T_ (kg ha^–1^)	TR (%)	TR_G_ (%)
2019	IS	CK	4739 ± 855b	1851 ± 291c	46.7 ± 5.8b	67.4 ± 5.2b
		D40	6756 ± 730a	2266 ± 83b	54.4 ± 9.7a	74.5 ± 7.1a
		D60	6358 ± 722a	2722 ± 84a	55.4 ± 5.8a	77.6 ± 9.2a
		D80	5802 ± 863ab	2348 ± 257b	55.4 ± 9.6a	68.4 ± 3.2b
	SS	CK	4050 ± 282a	1735 ± 149b	46.4 ± 3.7a	52.4 ± 3.1b
		D40	3893 ± 614a	2067 ± 357ab	49.4 ± 1.8a	60.4 ± 7.5ab
		D60	4457 ± 797a	2241 ± 274a	47.9 ± 4.3a	62.5 ± 3.2a
		D80	4897 ± 199a	2141 ± 267ab	47.2 ± 4.2a	62.4 ± 6.1a
2020	IS	CK	4324 ± 503b	2034 ± 149b	47.6 ± 5.9b	64.4 ± 2.1b
		D40	5544 ± 1121ab	2307 ± 191ab	51.4 ± 6.5a	73.6 ± 1.5a
		D60	6549 ± 1278a	2623 ± 105a	54.1 ± 2.9a	72.4 ± 6.3a
		D80	5536 ± 315ab	2125 ± 108b	53.4 ± 3.8a	64.6 ± 1.4b
	SS	CK	3602 ± 706b	1836 ± 257a	41.4 ± 4.7a	49.4 ± 4.1b
		D40	3926 ± 249ab	1992 ± 316a	43.8 ± 3.8a	57.3 ± 3.8a
		D60	4773 ± 423a	2216 ± 208a	42.4 ± 3.1a	61.4 ± 4.2a
		D80	4872 ± 656a	2203 ± 467a	42.6 ± 1.5a	60.4 ± 1.2a

			***F*-value**	***F*-value**	***F*-value**	***F*-value**

Year (Y)			0.02	12.9**	0.5	6.0*
Plant pattern (P)			2.6	33.5**	25.8**	25.9**
Treatment (T)			7.6**	38.6**	4.7*	4.8**
Y*P			34.2**	23.6**	28.2**	0.16
Y*T			4.6*	0.39	1.1	0.61
P * T			0.8	4.4*	2.1	4.4*
Y * P * T			4.9*	2.5	0.32	0.6

DM_A_, the soybean total dry matter accumulation from the R1 stage to the R8 stage; VDM_T_, the amount of soybean dry matter transferred into grains from the R4 stage to the R8 stage; TR, the dry matter transportation ratio of VDM_T_ to the dry matter of the vegetative organ at the R4 stage; TR_G_, the grains contribution ratio of VDM_T_. Different letters indicate significant differences between treatments at 0.05 probability levels by Fisher LSD multiple comparisons in the same planting pattern. * indicates statistical significance at 0.05 probability levels (p < 0.05) and ** indicates statistical significance at 0.01 probability levels (p < 0.01).

**FIGURE 6:**
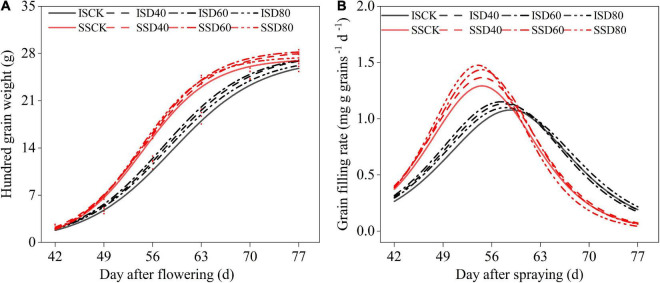
Spraying DA-6 affects the grain filling process of soybean. **(A)** Hundred-grain weight fit curve of soybean. **(B)** Grain filling rate fit curve of soybean. Black and red lines indicate relay intercropping soybean and monoculture soybean, respectively. The solid line, scribe line, dashed-dotted line, and dotted line mean the CK, D40, D60, and D80 treatment, respectively.

The grain-filling processes of all treatments fitted the logistic growth curve equations (Figure 6). The T_max_ and D were independent with the DA-6 treatments, T_max_ and D were higher in IS than in SS (Table 2). The D was about 7 days longer in IS than in SS. The W_max_, V_max_, and V_*mean*_ were increased by DA-6 treatments, and W_max_, V_max_, and V_mean_ were higher in SS than in IS. The V_max_ and V_mean_ were peaked at D60 in both IS and SS. In D60 treatment, the V_mean_ and V_max_ increased by 6.5% and 6.5% in IS and 5.7% and 4.3% in SS compared with the corresponding CK.

**TABLE 2:** Spraying DA-6 affect the grain filling parameters of soybean.

Plant pattern	Treatment	T_max_ (day)	W_max_ (mg grain^–1^)	V_max_ (mg grain^–1^ day^–1^)	V_mean_ (mg grain^–1^ day^–1^)	D (day)
IS	CK	58.8 ± 0.33a	13.6 ± 0.51a	1.08 ± 0.02b	0.31 ± 0.01b	37.9 ± 0.1a
	D40	57.8 ± 0.12a	14.1 ± 0.1a	1.13 ± 0.01a	0.33 ± 0.01a	37.2 ± 0.2a
	D60	57.4 ± 0.54a	14.0 ± 0.18a	1.15 ± 0.01a	0.33 ± 0.01a	37.6 ± 0.5a
	D80	58.2 ± 0.34a	13.8 ± 0.42a	1.11 ± 0.04ab	0.32 ± 0.01ab	37.6 ± 0.8a
SS	CK	54.6 ± 0.16a	13.6 ± 0.07b	1.38 ± 0.01b	0.35 ± 0.01a	30.8 ± 0.2a
	D40	54.7 ± 0.48a	41.2 ± 0.04a	1.37 ± 0.04b	0.36 ± 0.01a	31.0 ± 1.0a
	D60	54.6 ± 0.15a	14.2 ± 0.08a	1.44 ± 0.01a	0.37 ± 0.01a	30.8 ± 0.6a
	D80	54.1 ± 0.12a	13.8 ± 0.1b	1.48 ± 0.03a	0.36 ± 0.01a	29.8 ± 0.7a

		***F*-value**	***F*-value**	***F*-value**	***F*-value**	***F*-value**

Plant pattern (P)		716**	72.6**	757**	530**	219**
Treatment (T)		6.5*	10.4**	23.9**	38.6**	2.2
P*T		6.1*	5.7*	12.9**	5.7*	2.3

*T_max_, time reaching a maximum grain-filling rate; W_max_, the weight of maximum grain-filling rate; V_max_, maximum grain-filling rate; V_mean_, mean grain-filling rate; D, active grain-filling period.*

* indicates statistical significance at 0.05 probability levels (p < 0.05) and ** indicates statistical significance at 0.01 probability levels (p < 0.01).

### DA-6 Increases Soybean Pod Number and Yield

The soybean pod number, hundred-grain weight, and grain yields were significantly (*p* < 0.05) increased by DA-6 treatments, grain yield peaked at D60 in both IS and SS (Table 3). Independent with DA-6 treatments, the grain per pod was higher in IS than in SS. In D60 treatment, the soybean grain yield increased by 36.7–38.4% in IS and 21.7–26.6% in SS compared with the corresponding CK. The pod number was highest at D60 in IS. In D60 treatments, the pod number increased by 30.1–36.8% compared with corresponding CK. The pod number in SS peaked at D60 and D80 in 2019 and 2020, respectively. The pod number at D60 and D80 were increased by 13% and 6.3% compared with corresponding CK in 2019 and 2020, respectively. The hundred-grain weight peaked at D60 in both IS and SS. In D60 treatment, the hundred-grain weight was increased by 4.5–6.7% in IS and 3.6–5.6% in SS compared with the corresponding CK.

**TABLE 3:** Spraying DA-6 affects the yield and yield components of soybean.

Year	Planting patterns	Treatment	Pod number (10^4^ no. ha^–1^)	Grains per pod (Grains pod^–1^)	Hundred-grain weight (g)	Yield (kg ha^–1^)
2019	IS	CK	397 ± 78 b	1.59 ± 0.15 a	22.98 ± 1.64 b	1345 ± 298 b
		D40	458 ± 61 b	1.50 ± 0.15 a	23.73 ± 1.03 a	1504 ± 356 ab
		D60	543 ± 79 a	1.61 ± 0.11 a	24.52 ± 1.82 a	1861 ± 452 a
		D80	465 ± 65 ab	1.52 ± 0.14 a	23.14 ± 0.83 ab	1677 ± 348 ab
	SS	CK	469 ± 54 ab	1.50 ± 0.14 a	25.51 ± 1.3a	1712 ± 301 b
		D40	448 ± 59 b	1.48 ± 0.13 a	26.11 ± 0.77 a	1892 ± 435 ab
		D60	510 ± 53 ab	1.51 ± 0.16 a	26.42 ± 1.09 a	2167 ± 424 a
		D80	530 ± 46 a	1.55 ± 0.13 a	26.11 ± 0.95 a	1751 ± 368 b
2020	IS	CK	502 ± 48 c	1.54 ± 0.17 a	20.32 ± 0.92 b	1452 ± 237 b
		D40	583 ± 58 b	1.51 ± 0.17 a	20.43 ± 1.48 b	1859 ± 256 a
		D60	655 ± 49 a	1.57 ± 0.17 a	21.24 ± 1.04 a	1985 ± 356 a
		D80	515 ± 53 c	1.52 ± 0.16 a	19.88 ± 0.94 b	1525 ± 277 b
	SS	CK	617 ± 56 ab	1.46 ± 0.14 a	22.93 ± 1.79 b	1828 ± 128 b
		D40	580 ± 61 b	1.43 ± 0.14 a	23.73 ± 2.58 a	1855 ± 313 b
		D60	656 ± 48 a	1.49 ± 0.18 a	24.21 ± 1.91 a	2224 ± 334 a
		D80	616 ± 81 ab	1.49 ± 0.17 a	21.95 ± 3.28 c	2081 ± 495 a

			***F*-value**	***F*-value**	***F*-value**	***F*-value**

Year (Y)			78.43**	7.34**	325.57**	3.34
Planting patterns (P)			9.07**	21.16**	247.87**	6.98**
Treatment (T)			10.17	6.1**	13.69**	4.69**
Y * P			1.41	1.23	0.81	0
Y* T			1.32	0.16	2.52	0.36
P * T			5.12**	2.75*	0.29	0.15
Y* P * T			0.11	1.02	2.11	1.05

Different letters indicates significant differences between treatments at 0.05 probability levels by Fisher LSD multiple comparisons in the same planting pattern. * indicates statistical significance at 0.05 probability levels (p < 0.05) and ** indicates statistical significance at 0.01 probability levels (p < 0.01).

## Discussion

### DA-6 Increased the Soybean Leaf Photosynthetic Capacity to Promote Pod Formation During the Flowering-Podding Stage

The number of pod per plant, number of grains per plant, and hundred-grain weight were positively related to the soybean grain yield (Liu et al., 2019). Results in this experiment showed that the better-promoted effect of D60 on soybean pod number and hundred-grain weight resulting in the soybean yield was increased by 36.7–38.4% in IS and 21.7–26.6% in SS, and the soybean grains per pod showed no significant change (Table 3). The process of flower development into mature pods during the flowering and podding stage determines the number and quality of the soybean pod. Nutrition competition among sourced and sink organs caused flowers and pod abortion and abscission, decreasing soybean pod number and grain yield (Raza et al., 2020). Nitrogen application at the R1 stage increased the soybean grain yield by promoting biomass accumulation during the soybean reproductive growth stage (Zhou H. et al., 2019). Similarly, results in this experiment showed that the DA-6 significantly (*p* < 0.05) increased the soybean aboveground dry matter (Figure 5), and the yield and yield components of soybean (Table 3). The yield increase ratio and pod number increase ratio of soybeans were higher in IS than in SS, which was correlated with higher DM_A_ and VDM_T_ in IS than in SS (Table 1). Much of the yield variation of grain crops, including soybean, was associated with the variation in pod number per plant, hundred-grain weight, and seeds per plant (Egli, 2015). Similarly, Liu et al. (2019) found that the application of DA-6 increased the soybean podding set ratio by promoting biomass accumulation. The significant increase in soybean biomass accumulation resulted in a significant increase in soybean pod number in IS. From the results that the dry matter weight in IS was rapidly increased after spraying with DA-6, we suggested that the effect of DA-6 treatment on promoting the soybean after-flower growth was higher in IS than in SS. Previous studies found that after the maize has been harvested, the compensatory photosynthetic growth soybean in later periods alleviates the soybean flowers and pod abortion and abscission caused by insufficient assimilate accumulation in relay intercropped soybeans (Fan et al., 2019). We suggested that the application of DA-6 promotes the compensatory growth of relay intercropped soybean after the restoration of canopy illumination, thereby increasing the synthesis and accumulation of carbohydrates and promoting pod formation.

Leaves were essential photosynthetic organs, where the dry matter accumulation of plants depended on the capacity of the leaves to capture the light energy and convert it to chemical energy (Ba et al., 2020). The photosynthetic characteristics of crops are closely related to the photosynthesis area, photosynthesis efficiency, and photosynthesis time and are essential determinants of dry matter production in soybean (An et al., 2019). In this experiment, D60 increased the soybean LAI (Figure 2) to increase the canopy photosynthesis area, which may increase the amount of intercepted photosynthetically active radiation and the energy transformation capacity of the canopy. Moreover, further analyzing the variation in soybean leaf number and average leaf area showed that the leaf number significantly increased in IS and the change in soybean leaf number in SS showed no significant difference (Supplementary Figure 1). The increase in soybean leaf number in IS was consistent with Zhou T. et al. (2019), who found that the intercropped soybeans produced abundant newly trifoliate leaves after the maize harvest since the recovery of canopy light condition. We suggested that DA-6 increased soybean LAI primarily by promoting the leaf number of relay intercropped soybean and the average leaf area of monoculture soybean.

Photosynthesis is a complex process in plant chloroplasts that convert light into chemical energy through photosynthetic pigments (Yao et al., 2017). DA-6 significantly (*p <* 0.05) increased the soybean total chlorophyll content and Pn of soybean after the R1 stage (Figure 3 and Supplementary Figure 2), which were crucial photosynthesis parameters that reflect the efficiency of the crop synthesis carbohydrate. The enhancement of soybean chlorophyll content in IS was better than in SS, which contributed to the significant change in Pn in IS after spraying with DA-6. The increase in chlorophyll content and Pn of the relay intercropped soybean after the restoration of soybean canopy illumination is consistent with Fan et al. (2018). Wen et al. (2019) also found that the application of tertiary amine bioregulators promoted photosynthesis pigment synthesis and larger chloroplasts, increasing soybean photosynthesis rates and carbohydrate concentrations. Thus, we suggested that the application of DA-6 showed a better promoting effect on chlorophyll in IS than in SS, which results in the higher Pn in IS after the R1 stage.

Diethyl aminoethyl hexanoate showed a higher promoting effect on the photosynthesis efficiency of relay intercropped soybean, which contributed to the growth of relay intercropped soybean after the flowering stage. The application of DA-6 promoting the soybean photosynthetic function after-flower stage alleviates defective material and energy accumulation of soybean and improves pod and grain formation. DA-6 significantly (*p <* 0.05) increased the photosynthesis area and net photosynthesis rate of soybean to improve the carbohydrate synthesis of the leaves during the soybean flowering and podding stage, ultimately promoting flower and pod formation increasing soybean pod number. And, the increase in soybean pod number enhanced the ability of sink organs, which may promote the biomass translocation from source organs and contribute to the soybean grain yield formation.

### DA-6 Delayed the Leaf Senescence to Improve the Soybean Grain-Filling Process at the Later Stages

Soybean grain number and weight were essential components of soybean yield and were influenced by the soybean grain-filling process. This experiment showed that DA-6 increased the hundred-grain weight of relay intercropped soybean and maintained the stability of seed number per pod when the pod number was significant at *p <* 0.05 (Table 3). The grain-filling rate and duration time were essential indicators that reflect the ability of the soybean grain-filling process. TR and TR_G_ were essential indicators to reflect the ability of the soybean to translocate the carbohydrate source to sink organs and its contribution to grain formation (Luo et al., 2020; Kelly et al., 2021). Results in this experiment showed the V_max_ and V_mean_ values of soybean were increased in DA-6 treatments (Table 2), which was correlated with higher DM_A_ and VDM_T_ in DA-6 treatments (Table 1). He et al. (2020) also proved that increasing V_max_ and V _mean_ optimized the crop grain-filling process and weight of wheat grains. And the *D* values were about 7 days longer in IS than in SS, which may suggest that the soybean had a longer active grain-filling day in IS than in SS. An extended period of photosynthesis during the leaf senescence process, which always happens during the grain-filling stage, is essential for promoting soybean grain-filling processes (Egli, 2010). We suggested that DA-6 promoted the biomass translocation into grains and increased the V_max_ and V_mean_ of relay intercropped soybean to promote soybean grains formation. And the increase in pod number may benefit the biomass translocation since the pod was an essential sink organ that affects the transportability of assimilates. Thus, the grains per pod were stable while the pod number and hundred-grain weight were significantly (*p* < 0.05) increased by DA-6 treatments in IS.

The premature leaves senescence leads to the empty pod during the grain filling process, which correlated with the photosynthesis pigments degradation and photosynthesis rate decrease (Chen et al., 2018). The premature leaves are accompanied by the accumulation of generation of reactive oxygen (ROS) in cells, which initiates membrane lipid peroxidation and directly damages the normal function of chloroplast by reducing the chlorophyll content and photodamage to PSII and D1 proteins (Jiang, 2021). The content of H_2_O_2_ and O_2_^–^ in soybean leaves was an essential indicator of ROS accumulation, and the MDA content reflects the levels of lipid peroxidation (Yao et al., 2017). Plants effectively convert toxic H_2_O_2_ to water and nontoxic oxygen *via* the CAT, POD, and the ascorbate-glutathione cycle (including APX, GR, GSSG, and GSH) (Luo et al., 2020). DA-6 significantly (*p <* 0.05) decreased the H_2_O_2_ and O_2_^–^, and MDA content in soybean leaves indicated that the application of DA-6 alleviated the damage caused by ROS accumulation and leaves in SS earlier senescence than in IS (Figure 4). And the results that DA-6 significantly (*p <* 0.05) enhanced the activities of SOD, POD, CAT, and APX and increased the content of GSSG and GSH also proved that the application of DA-6 delayed the leaf senescence of soybean leaves (Figure 4 and Supplementary Figure 3). The effect of DA-6 delay leaf senescence was consistent with Fu et al. (2011), who showed that the application of DA-6 increased the enzyme activity of SOD, CAT, and APX to reduce the O_2_^–^ and H_2_O_2_ production, eventually alleviating the lipid peroxidation damage under chilling stress. We suggested that DA-6 increased the activity of the antioxidant defense systems to delay the leaf senescence and prolonged the soybean grain-filling stage to maintain the biomass supply during the soybean grain-filling stage, which benefit the biomass transfer into grains and increased the grain-filling process and the soybean grains weight and number. And the effect of DA-6 on the delayed senescence of soybean leaves during the grain-filling stage was performing better in IS than in SS. Thus, DA-6 can increase the hundred-grain weight of soybean and maintain the stability of grain number per pod under the situation that the pod numbers were significantly increased after spraying with DA-6.

## Conclusion

DA-6 treatments increased the soybean LAI by promoting the newly born leave growth in IS and expanding the average leave area in SS, and the LAI increase ratio in IS was higher than in SS. Although the chlorophyll content and net photosynthesis were both increased by DA-6 treatments in IS and SS, the effect of DA-6 on the delayed senescence of soybean leaves during the grain-filling stage was performing better in IS than in SS. The D was about 7 days longer in IS than in SS. And the increase in DMT and TRG was higher in IS than in SS, which may partly explain why the increase in V_mean_ and V_max_ was higher in IS. The grains per pod were stable while the pod number and hundred-grain weight were significantly (*p* < 0.05) increased by DA-6 treatments in IS. The higher effect of DA-6 on improving soybean photosynthesis area and net photosynthesis of IS results in the pod number and hundred-grain weight at D60 treatment were increased by 30.1–36.8% and 4.5–6.7% in IS and 6.3–13% and 3.6–5.6% in SS. Thus, the soybean grain yield was peaked at D60 treatment and increased by 36.7–38.4% in IS and 21.7–26.6% in SS.

## Data Availability Statement

The original contributions presented in the study are included in the article/Supplementary Material, further inquiries can be directed to the corresponding author.

## Author Contributions

TY and WY designed the research. KL, XY, SL, PC, and QD performed the experimental work and data analysis. BZ, YW, and XW provided helpful suggestions for the data analysis and manuscript revision. KL wrote the manuscript. All authors read and approved the final manuscript.

## Conflict of Interest

The authors declare that the research was conducted in the absence of any commercial or financial relationships that could be construed as a potential conflict of interest.

## Publisher’s Note

All claims expressed in this article are solely those of the authors and do not necessarily represent those of their affiliated organizations, or those of the publisher, the editors and the reviewers. Any product that may be evaluated in this article, or claim that may be made by its manufacturer, is not guaranteed or endorsed by the publisher.

## Abbreviations

IS: maize-soybean relay strip intercropping system
SS: monoculture soybean
DA-6: diethyl aminoethyl hexanoate
CK: 0 mg L^–1^ of DA-6
D40: 40 mg L^–1^ of DA-6
D60: 60 mg L^–1^ of DA-6
D80: 80 mg L^–1^ of DA-6
PAR: photosynthetically active radiation
LAI: leaf area index
R1: the beginning flower stage
R2: blooming flower stage
R3: beginning pod stage
R4: blooming pod stage
R5: beginning seed stage
R6: grain-filling stage
SOD: superoxide dismutase
POD: peroxidase
CAT: catalase
O_2_^–^: superoxide anion
H_2_O_2_: hydrogen peroxide
MDA: malondialdehyde
APX: ascorbic acid peroxidase
GR: glutathione reductases
GSH: glutathione
GSSG: oxidized glutathione
DM_A_: soybean total dry matter accumulation from the R1 stage to the R8 stages
VDM_T_: amount of soybean dry matter transfer into grains from the R4 stage to the R8 stage
TR: dry matter transportation ratio of VDM_T_ to the dry matter of soybean vegetative organ at the R4 stage
TR_G_: the grains contribution ratio of VDM_T_
T_max_: the time required to reach the maximum grain-filling rate
T: the duration time when W was achieved at the 99% of A
V_max_: maximum grain-filling rate
V_mean_: mean grain-filling rate
W_max_: grain weight at maximum grain-filling rate
D: active grain-filling period and defined as the period when W was between 5 and 95% of A.
